# Supplementary material for: Lost and Found: Re-searching and Re-scoring Proteomics Data Aids Genome Annotation and Improves Proteome Coverage
Source: mSystems. 2020 Oct 27;5(5):e00833-20. doi: 10.1128/mSystems.00833-20 (PMC7593589; doi:10.1128/mSystems.00833-20)
Supplement: TABLE S1 [file mSystems.00833-20-st001.pdf]

Feature name	Description
MS-GF+ feature set	
RawScore	Raw match score of MS-GF+
DeNovoScore	Maximum possible raw match score to this spectrum
ScoreRatio	RawScore divided by DeNovoScore
Energy	Difference between RawScore and DeNovoScore
lnEValue	Negative MS-GF+ E Value, logged
lnSpecEValue	Negative MS-GF+ Spectral E Value, logged
IsotopeError	Number of additional neutrons in peptide
lnExplainedIonCurrent	Summed intensity of identified fragment ions, divided by that of all fragment ions, logged
lnNTermIonCurrentRatio	Summed intensity of identified Nt fragments divided by that of all identified fragments, logged
lnCTermIonCurrectRatio	Summed intensity of identified Ct fragments, divided by that of all identified fragments, logged
lnMS2IonCurrent	Summed intensity of all observed fragment ions, logged
ExpMass	Experimental peptide mass
CalcMass	Calculated peptide mass
PepLen	Peptide length
dM	Difference between theoretical and experimental mass
absdM	Absolute value of the difference between theoretical and experimental mass
MeanErrorTop7	Mean of mass errors of the seven fragment ion peaks with the highest intensities
sqMeanErrorTop7	Squared MeanErrorTop7
StdevErrorTop7	Stdev of mass errors of the seven fragment ion peaks with the highest intensities
ChargeN	Boolean, peptide charge is N
enzN	Boolean, N-terminal agrees with enzymatic cleavage rules
enzC	Boolean, C-terminal agrees with enzymatic cleavage rules
enzInt	Number of internal cleavage sites
Auxiliary features	
spec_pearson_norm[a]	Pearson correlation log normalized spectrum
dotprod_norm[a]	Dot product log normalized spectrum
spec_mse[a]	Mean squared error normalized spectrum
dRT	Absolute difference experimental RT and ELUDE predicted RT (in mins)
RK	Number of Arg and Lys in peptide
lnYfrac	Identified Y-ions (0.01 if none) divided by number of theoretical Y-ions, logged
lnBYfrac_ms2pip	Fraction of B/Y-ions agreeing to MS^{2}PIP-predicted absence/presence of B/Y-ion, logged
lnCovPos	Covered positions divided by peptide length – 1, logged
PathLen	Longest consecutive path BY-ions
BY	Number of matched B/Y-ions
BY_pred5perc	Number of matched B/Y-ions explaining at least 5% of intensity in MS^{2}PIP-predicted spectrum

Combined feature set	
MS-GF+ features	All features described above
Extra features	All features described above
