# Supplementary material for: Lost and Found: Re-searching and Re-scoring Proteomics Data Aids Genome Annotation and Improves Proteome Coverage
Source: mSystems. 2020 Oct 27;5(5):e00833-20. doi: 10.1128/mSystems.00833-20 (PMC7593589; doi:10.1128/mSystems.00833-20)
Supplement: TABLE S2 [file mSystems.00833-20-st002.pdf]

Feature Set	Level	First search	Second search	Third search
MS-GF+	Peptide	25,895	16,840	4,925
	Spectra	574,182	131,456	15,463
Auxiliary	Peptide	25,494	17,851	6,099
	Spectra	561,772	142,313	21,673
Combined	Peptide	26,440	19,283	7,585
	Spectra	578,708	156,960	27,465
