# Supplementary material for: Lost and Found: Re-searching and Re-scoring Proteomics Data Aids Genome Annotation and Improves Proteome Coverage
Source: mSystems. 2020 Oct 27;5(5):e00833-20. doi: 10.1128/mSystems.00833-20 (PMC7593589; doi:10.1128/mSystems.00833-20)
Supplement: TEXT S1 [file mSystems.00833-20-s0001.docx]

***Supplemental methods***

**Ribosome profiling data analysis**

Previously published ribosome profiling data from *S*. Typhimurium SL1344 (GEO accession GSE91066, (1)) was re-analyzed. Raw sequencing data of polysome and monosome samples were concatenated and adapter sequences were removed from the reads using fastx_clipper and reads aligning onto rRNA and tRNA sequences were discarded. The remaining reads were aligned to the *S*. Typhimurium SL1344 genome (chromosome and plasmids) using STAR allowing only for uniquely mapped reads. Ribosome occupancy positions were assigned based on the plastid predicted p-site offsets using the 3’ end for p-site assignment (13). Reads with a length greater than 42 nt were low abundant and of low quality and were therefore discarded.

**LC-MS/MS analysis**

Samples were analyzed by LC-MS/MS using an UltiMate 3000 RSLC nano HPLC (Dionex) connected in-line to a Q-Exactive HF mass spectrometer (Thermo Fisher Scientific Inc.). Samples were separated on a 40 cm column packed in the needle (produced in-house, 75 μm I.D. ×400 mm, 1.9 μm beads C18 Reprosil-HD, Dr. Maisch) using a non-linear 150 min gradient of 2-56% solvent B’ (0.1% formic acid (FA) in water/ACN, 20/80 (v/v)) at a flow rate of 250 nL/min. This was followed by a 10 min wash reaching 99% solvent B’ and re-equilibration with solvent A (0.1% FA in water). Column temperature was kept constant at 50°C (CoControl 3.3.05, Sonation). The mass spectrometer was operated in data-dependent, positive ionization mode, automatically switching between MS and MS/MS acquisition for the 16 most abundant peaks in a given MS spectrum. The source voltage was set to 3.5 kV and the capillary temperature was 250°C. One MS1 scan (*m*/*z* 375-1500, AGC target 3E6 ions, maximum ion injection time of 45 ms) acquired at a resolution of 60,000 (at 200 *m*/*z*) was followed by up to 16 MS/MS scans (resolution 15,000 at 200 *m*/*z*) of the most intense ions fulfilling predefined selection criteria (AGC target 1E5 ions, maximum ion injection time of 60 ms, isolation window of 1.5 m/z, fixed first mass of 145 m/z, spectrum data type: centroid, under fill ratio 2%, intensity threshold 1.3E4, exclusion of unassigned, singly charged precursors, peptide match preferred, exclude isotopes on, dynamic exclusion time of 12 s). The HCD collision energy was set to 32% Normalized Collision Energy and the polydimethylcyclosiloxane background ion at 445.12002 Da was used for internal calibration (lock mass).

**Post-processing proteogenomics pipeline**

The pipeline itself performs consecutive

*First round search and post-processing*

Thermo RAW files were converted to peak lists using ThermoRawFileParser (2). Resulting MGF (Mascot generic format) files were searched against the constructed peptide database using MS-GF+ (v2019.04.18) with enzymatic cleavage disabled. Methionine oxidation was set as fixed and the top 3 PSMs were considered per scan. The used command line is:

java -jar MSGFPlus.jar -decoy ‘decoy_’ -tda 0 -t 20 ppm -ti -1,3 -n 3 -m 3 -inst 3 -e 9 -maxCharge 4 -minLength 7 -addFeatures 1 -mod Mods.txt <MGF_input> pepLib.fasta

Note that the used search database (pepLib.fasta) is composed of tryptic target and decoy (prefix ‘decoy_’). Full peptides are searched by ‘-e 9’ (no cleavage). Using the ‘msgf2pin’ function provided by Percolator-converter package, a Percolator (3) tab-delimited input file was extracted from the MS-GF+ mzid result files, delivering in total 22 features per PSM (Table S1). For each sample, the retention time (RT) of the top 1,000 ranked unique peptide sequences (highest MS-GF+ score) was used to train a peptide RT model by ELUDE (v3.02.1) (4). This model was used to predict the RT for all matched peptides and the absolute deviation of the predicted and experimental RT (in mins) was used as additional scoring feature in the auxiliary feature set (Table S1). In addition, the reScore algorithm (5) was used to compare the fragment peak intensities of empirical spectra with those predicted MS²PIP (algorithm v20190312, HCD trained model v20190107) (6). Of the resulting features, ‘spec_pearson_norm’, ‘dot_prod_norm’ and ‘spec_mse’ were used in the auxiliary feature set given their relatively high weights in Percolator learning. The auxiliary feature set was further expanded with six features, including the number of Arg/Lys residues (i.e. reflecting trypsin missed cleavages) in the peptide, as well as features reflecting the number of matched b/y-ions (Table S1). Using the MS-GF+, auxiliary and combined feature sets, Percolator (v3.02.1) (3) was used to re-score PSMs for every MS-GF+ search.

percolator –override -U -v 2 -Y -w <weights_output> -m <PSM_target_output> -M <PSM_decoy_output> <pin_input>

The feature weights of the combined feature set were displayed in Figure S2. Subsequently, the obtained Percolator recalibrated PSM scores were used for class-specific confidence estimation for annotated (Ensembl) and unannotated peptides. To this end Percolator was ran without any learning iterations (“--max-iterations 0”) on concatenated target and decoy PSMs with initial weight assigned to the recalibrated Percolator score. Resulting peptide identifications were filtered at a 1% peptide *Q*-value for annotated peptides and 5% peptide *Q*-value for unannotated peptides.

*Iterative searches for identification of co-fragmented peptides*

To identify co-fragmented peptides in a MS² spectrum we used an iterative search strategy resembling the rationale followed in the reSpect algorithm (7). For MS² spectra with an assigned PSM (Q-value ≤ 0.01) in the prior search, matching (tolerance ≤ 0.02 *m*/*z*) b/y-ions were omitted from the spectrum (including double charged fragment ions and/or ions with neutral losses). Spectra were re-searched for charge states 2+ and 3+, duplicating each spectra, using MS-GF+ with parameters as described above, except setting a wider precursor mass tolerance of 3.1 Da and disabling isotope peak errors (‘-t 3.01Da -ti 0,0’). Such wider tolerance allows identification of non-monoisotopic peptides in the isolation window (7). PSM feature generation and Percolator post-processing was performed as described above, except that the peptide RT model trained in the first search by ELUDE was used.

**MaxQuant search**

Proteomics data was searched in parallel by MaxQuant (version 1.6.7.0, (8)) against the Ensembl *S.* Typhimurium SL1344 annotated proteome (genome assembly ASM21085v2). Methionine oxidation was set as fixed modification and matching-between-runs as well as the LFQ algorithm were enabled. MaxQuant was ran twice, the only difference being the second peptide search option, which performs an iterative search for co-fragmenting peptides (9).

**References supplemental methods**

1. Ndah E, Jonckheere V, Giess A, Valen E, Menschaert G, Van Damme P. 2017. REPARATION: ribosome profiling assisted (re-)annotation of bacterial genomes. Nucleic Acids Res 45:e168.

2. Hulstaert N, Shofstahl J, Sachsenberg T, Walzer M, Barsnes H, Martens L, Perez-Riverol Y. 2020. ThermoRawFileParser: Modular, Scalable, and Cross-Platform RAW File Conversion. J Proteome Res 19:537-542.

3. Kall L, Canterbury JD, Weston J, Noble WS, MacCoss MJ. 2007. Semi-supervised learning for peptide identification from shotgun proteomics datasets. Nat Methods 4:923-5.

4. Moruz L, Tomazela D, Kall L. 2010. Training, selection, and robust calibration of retention time models for targeted proteomics. J Proteome Res 9:5209-16.

5. Silva ASC, Bouwmeester R, Martens L, Degroeve S. 2019. Accurate peptide fragmentation predictions allow data driven approaches to replace and improve upon proteomics search engine scoring functions. Bioinformatics doi:10.1093/bioinformatics/btz383.

6. Degroeve S, Martens L. 2013. MS2PIP: a tool for MS/MS peak intensity prediction. Bioinformatics 29:3199-203.

7. Shteynberg D, Mendoza L, Hoopmann MR, Sun Z, Schmidt F, Deutsch EW, Moritz RL. 2015. reSpect: software for identification of high and low abundance ion species in chimeric tandem mass spectra. J Am Soc Mass Spectrom 26:1837-47.

8. Cox J, Mann M. 2008. MaxQuant enables high peptide identification rates, individualized p.p.b.-range mass accuracies and proteome-wide protein quantification. Nat Biotechnol 26:1367-72.

9. Cox J, Neuhauser N, Michalski A, Scheltema RA, Olsen JV, Mann M. 2011. Andromeda: a peptide search engine integrated into the MaxQuant environment. J Proteome Res 10:1794-805.
